# The Influence of Examiner Experience on Inter- and Intra-Rater Agreement in Dental Restorations Identification on Three-Dimensional Digital Models

**DOI:** 10.3390/medicina61122135

**Published:** 2025-11-29

**Authors:** Marion Nigoghossian, Meda-Romana Simu, Bogdan Culic, Sorina Sava, Henri Bouteiller, Carina Culic, Iulia Clara Badea, Ondine Patricia Lucaciu, Aranka Ilea, Ioana Porumb

**Affiliations:** 1 Faculty of Dental Medicine, Iuliu Hațieganu University of Medicine and Pharmacy, 8 Victor Babes Str., 400012 Cluj-Napoca, Romania; marion.audr.nigoghossian@elearn.umfcluj.ro (M.N.); bouteiller.henri.francois@elearn.umfcluj.ro (H.B.); 2 Pediatric Dentistry Department, Iuliu Hațieganu University of Medicine and Pharmacy, 31 Avram Iancu Str., 400083 Cluj-Napoca, Romania; 3 Dental Propedeutics and Aesthetics Department, Iuliu Hațieganu University of Medicine and Pharmacy, 32 Clinicilor Str., 400006 Cluj-Napoca, Romania; bculic@umfcluj.ro; 4 Dental Materials Department, Iuliu Hatieganu University of Medicine and Pharmacy, 31 Avram Iancu Str., 400083 Cluj-Napoca, Romania; savasorina@elearn.umfcluj.ro; 5 Odontology Department, Iuliu Hațieganu University of Medicine and Pharmacy, 33 Calea Motilor, 400001 Cluj-Napoca, Romania; 6 Preventive Dentistry Department, Iuliu Hațieganu University of Medicine and Pharmacy, 31 Avram Iancu Str., 400083 Cluj-Napoca, Romania; iulia.badea@umfcluj.ro (I.C.B.); porumb.ioana@umfcluj.ro (I.P.); 7 Oral Health Department, Iuliu Hațieganu University of Medicine and Pharmacy, 15 Victor Babes Str., 400012 Cluj-Napoca, Romania; patricia.lucaciu@umfcluj.ro; 8 Oral Rehabilitation, Oral Health and Dental Office Management Department, Faculty of Dentistry, Iuliu Hatieganu University of Medicine and Pharmacy, 15 Victor Babes Str., 400012 Cluj-Napoca, Romania; aranka.ilea@umfcluj.ro

**Keywords:** three-dimensional imaging, preventive dentistry, dental restorations, artificial intelligence, dental caries diagnosis, oral, diagnostic imaging

## Abstract

*Background and Objectives*: In recent decades the prevalence of dental caries has continued to increase despite widespread access to modern dental care. This study aimed to evaluate the diagnostic relevance of differentiating healthy, carious, and restored dental structures using intraoral scans—a non-irradiating imaging technique. *Materials and Methods*: A cross-sectional reliability (diagnostic agreement) study was carried out. All 36 examiners underwent pre-calibration on the ICDAS scores recordings. They filled in ICDAS files for each randomly assigned patient through three different methods: clinical examinations and three-dimensional digital models obtained with two different intraoral scanners. Cohen’s weighted kappa test and Prevalence and Biased Adjusted Kappa (PABAK) were utilized to evaluate agreements. A corresponding *p*-value for agreement was computed for each agreement coefficient. *Results*: When ICDAS values recorded using intraoral scanners were compared for examiners with different experience levels, there was an inter-rater substantial agreement for all teeth (PABAK = 0.688; CI 95% = 0.344–0.894), but also for anterior teeth only, as well as for smooth surfaces only. Regardless of the examiner’s clinical experience, PABAK inter- and intra-rater agreement on fillings identification on three-dimensional digital models obtained by intraoral scanning were at least moderate in most cases. *Conclusions*: Three-dimensional digital models offer reliable diagnostic information, especially for experienced clinicians, supporting their use as a standardized tool in routine practice to obtain an accurate, dynamic view of patients’ caries status, although limitations such as controlled study conditions and variability in scanning algorithms across systems must also be acknowledged in daily dental diagnostics.

## 1. Introduction

Historically, dental caries have been known as the oldest and most predominant oral health problem, and it continues to represent a major public health issue [1,2]. In post-industrial nations, the decay prevalence rose sharply as wealth did and especially as processed sugar became more widely marketed. Carious lesions can be identified not only by clinical visual techniques but also by tactile examinations, often supplemented by imaging methods like radiography, which is the most widely used complementary diagnostic tool [3]. Early detection of dental caries increases the chances of proceeding to a non-invasive or minimally invasive treatment [4]. Detecting secondary caries (recurrent caries), requires routine monitoring, which can be quite challenging [4,5].

Accurate assessment of the number of caries and dental restorations can provide a snap-shot image of each patient’s current and past caries experience. Because diagnostic reproducibility depends not only on identifying lesions but also on consistently detecting existing fillings, reliable documentation of restorations becomes essential—particularly as digital methods increasingly support clinical decision-making. The International Caries Detection and Assessment System (ICDAS) aims to record existing restorations (codes 0–8) and classify the various stages of the carious process, from early visible enamel changes to extensive cavitation (codes 0–6 for the second figure of the ICDAS code assigned to each surface), including situations in which a surface cannot be examined (codes 96–99).

ICDAS visual inspection protocol requires examiners to first assess each clean and dry tooth surface to determine if it was intact, sealed, restored (if so, a code for the restoration material must be recorded), crowned, or absent [6]

This method was adapted precisely to target and assess caries in conjunction with restorations and sealants: ICDAS II [7]. Research has shown that the ICDAS II presents a fairly good reproducibility and accuracy for in vitro and in vivo detection of past caries experience and primary carious lesions at different levels in the progression of the disease [8]. In total, an ICDAS file assesses a maximum of 174 areas (plus 32 possibly exposed roots):

- Seven Surfaces for six upper Molars.
- Five Surfaces for
  ◾ Six upper Canines and Premolars;
  ◾ Six lower Canines and Premolars;
  ◾ Four upper Incisors.
- Four surfaces for four lower Incisors.
- Six surfaces for six lower Molars.

Besides clinical examination, equipment such as the DIAGNOdent laser fluorescence device helps to detect secondary caries beneath restorations by measuring bacterial fluorescence [9,10]. However, its accuracy may be affected by interference from amalgam restorations [5,11,12].

Caries experience detection is still an important challenge despite modern advancements [13]. In recent years, there has been a growing emphasis on the use of three-dimensional (3D) digital models to detect oro-dental diseases [14,15,16,17]. Intraoral scanning systems (IOS) combining optical caries detection are attracting particular attention from researchers. Intraoral scanning offers the advantage of capturing tissue geometry along with realistic colors (R, G, B) and fluorescence data, all of which can be visualized on 3D dental models. Intraoral scanners software is expected to be widely adopted for more effective management of carious disease through caries risk assessment, diagnostic, and monitoring. Dentists must maximize all the diagnostic opportunities offered by this method, thus allowing early therapeutic intervention because of the convincingly proven influence of deleterious habits on dental caries progression [18].

Michou et al. have published several articles on IOS, carious lesions assessment, and 3D models use for secondary caries detection, among which are in vitro and in vivo studies. For example, on 116 extracted teeth authors proved a correlation among secondary caries and the presence of gap measured with a three-dimensional (3D) intraoral scanner. Secondary caries detection and treatment decisions can be based on two criteria of a three-dimensional intraoral scanner on gap evaluation [14,15].

Therefore, clinical diagnosis based on color, translucency, and opalescence changes seem to be among the most accurate early-stage caries diagnosis. Yet, some articles about color perception showed a significant effect of assessor’s sex on visual color matching in dentistry [19].

The Decayed, Missing, and Filled Surface (DMF-S) index is a validated epidemiological measure of cumulative caries experience and is used to identify individuals and populations at increased risk for future caries development. Elevated DMF-S scores reflect a greater burden of past and present caries, which in turn signals a higher need for preventive strategies.

The medical literature demonstrates that individuals and populations with higher DMF-S indices benefit from caries-preventive interventions, particularly fluoride-based procedures. Randomized trials and systematic reviews showed that regularly repeated applications of topical fluoride gels, varnishes, and supplements are associated with significant reductions in DMF-T/DMF-S increments, especially in settings with high baseline caries burden as measured by DMF-S indices [20,21].

In recent years, several studies have demonstrated that AI-based systems can reliably detect and classify dental restorations, including fillings, crowns, implants, and other treatments, on two-dimensional radiological images (panoramic and bitewing radiographs) with high accuracy and sensitivity, as well as on 3D ones, meaning cone-beam computed tomography (CBCT) [22,23,24,25,26,27,28,29]. Recent deep learning models, such as Faster R-CNN, Mask DINO, and collaborative learning frameworks, have achieved mean average precision (mAP) values exceeding 0.90 and F1-scores above 0.91 for the detection and segmentation of dental fillings and other restorations on radiological images [22,26,28]. These systems can differentiate between restoration types and tooth categories, and their diagnostic performance is comparable to or exceeds that of experienced clinicians in controlled studies [23,24,27,28]. AI-driven tools also offer significant time efficiency and can reduce observer-dependent variability, support clinical workflow, and improve consistency in dental charting and treatment planning [28,30,31]. Some limitations of the previously mentioned research may be that all the examinations performed are based on irradiating detection methods and also that tooth-colored restorations are frequently misclassified or missed due to their radiographic and photographic similarity to natural tooth structure, resulting in lower sensitivity and precision compared to metallic restorations [24,32].

In the context of minimally invasive medicine and reduced exposure to X-rays, we wanted to see how relevant the differentiated recognition of healthy, carious, and filled dental structures based on intraoral scans, a non-irradiating method, can be. Therefore, our main research question is how easy it is to identify the number of ICDAS areas covered by fillings on 3D digital models as a reflection of past caries experience

The objectives of this study were to assess the intra-rater scores agreement for different examination methods, the inter-rater agreement for the same examination method, for all teeth, or for groups of teeth and groups of surfaces (smooth/pits and fissures) when identifying dental restorations. The aims were to evaluate the intra-rater and inter-rater agreement for dental restorations presence and the type of the used material: first ICDAS figure for cases where restorations were present (this first ICDAS score figure > 0 and < 9 as cumulative conditions).

In line with these objectives and to structure the evaluation of diagnostic reproducibility, we formulated the following hypotheses:

**H1:**  *When using the same examination method, examiners will demonstrate substantial intra-rater agreement in identifying the presence of restorations.*

**H2:**  *Inter-rater agreement for restoration detection will be comparable across examiners applying the same diagnostic method.*

**H3:**  *Agreement levels will differ between tooth groups and surface types, with smooth surfaces yielding higher agreement than pits-and-fissure surfaces.*

## 2. Materials and Methods

### 2.1. Study Design and Setting

A cross-sectional reliability (agreement) diagnostic study was carried out at the Faculty of Dentistry, Iuliu Hațieganu University of Medicine and Pharmacy, in Cluj-Napoca, Romania (UMFIH). This research received approval from the Ethics Committee of the “Iuliu Haţieganu” University of Medicine and Pharmacy (DEP125/20.04.2023). Prior to participation in the study, participants received thorough information about the research. All participants provided signed informed consent before being enrolled, which explicitly stated the freedom to withdraw from the study anytime and data management protocol, including anonymization and data deletion upon participant’s request.

### 2.2. Participants

The study involved 30 subjects (non-clinical population) randomly selected from a pool of 50 general dentistry interns and 78 third-year dental students who volunteered to take part. The requirements for participation as a patient included adult volunteers, students, and interns in general dentistry at the Department of Preventive Dentistry of Iuliu Hațieganu University of Medicine and Pharmacy, Cluj-Napoca, Romania. The sample size was selected according to the availability of the intraoral scanners, examiners, and volunteers. The statistical analysis unit was ICDAS-areas: up to 206 areas per patient, per examination for whose restorative and caries status examiners assigned two-digits codes: first digit 0 to 8 for restorations; second digit 0 to 6 for caries. Special codes 96 to 99 were assigned for surfaces that cannot be examined or were missing depending on the reason.

The study did not include children and patients with contraindications to professional cleaning.

At the beginning of each examination session, we used convenience sampling: the patients were conveniently assigned an examination category (clinical examination or one of the two intraoral scanners) and an examiner from a pool of 36 dental professionals aged between 22 and 46 years old divided into three proficiency levels: (1) eighteen third-year dental undergraduates; (2) ten general dentistry trainees (with 1–3 years’ practical experience); and (3) eight experienced dental professionals (with over 5 years’ clinical experience). The available examiners, at the beginning of each examination session, were given computer generated random numbers. For each patient, a random number was chosen to assign his/her corresponding examiner. The assessor was randomly chosen after eliminating raters who had previously examined the same patient (or their digital 3D models) within the past three months.

### 2.3. Diagnostic Methods

Patients were assessed using the ICDAS file methodology and/or intraoral scans after preparation for the study.

Prior to the examination, all teeth underwent thorough professional cleaning using a water-powder jet cleaner at 135° (autoclavable, Air flow → Handy 2+, EMS, Nyon, Switzerland) which contained sodium bicarbonate powder. Following this, powder remnants were eliminated by rinsing the teeth with a water spray for 5 s each.

#### 2.3.1. Visual Examination (ICDAS)

Examinations took place in a dental clinic with adequate illumination (25,000 lx of the dental unit lamp), utilizing an air syringe, a plane buccal mirror, and, if needed, a WHO (World Health Organization) periodontal probe. The examiner conducted an in vivo assessment of all teeth, initially under wet conditions and subsequently on surfaces which were air-dried for 5 s. The caries lesions and the existing restorations were classified based on primary coronal detection criteria as per the ICDAS II criteria [25,26,27] and assigned a two-digit code. The first digit indicates the tooth restoration, and codes range from 0 to 9. The second digit, coding for caries, ranges from 0 to 6.

All examiners underwent pre-calibration through training on the ICDAS clinical caries and restorations criteria alongside recording of caries experience clinical scores; this was overseen by an ICDAS-trained university lecturer. The training was provided hands-on by the same team of two university lecturers, who are both ICDAS-validated trainers. The caries assessment training included both an initial on-site ICDAS course that outlined the diagnostic criteria, as well as further hands-on instruction. During the hands-on training, five examinations of patients were performed (one clinical examination and two examinations performed using each intraoral scanner), and participants evaluated teeth that presented restorations and the caries lesions across all severity and cavitation levels. In case of raters’ disagreement, additional cases were added to improve the pre-calibration until at least 4 of 5 surfaces received the same ICDAS codes from all raters, meaning the calibration reliability values (intra-examiner kappa) were at least 0.8.

The examination conditions were similar (same dental units, pre-calibration of each intraoral scanner according to the manufacturer’s instructions using the calibration tool). All examiners had a 30 min training time for each scanner using the practice model provided by its manufacturer, including standardized scanning protocols: continuous horizontal scanning with minimal vertical rotation, starting from a defined location (the last molar of hemiarch 1 and, respectively, hemiarch 3), and following a prescribed path (occlusal surfaces first, then buccal and palatal/lingual surfaces) and post-scan verification and quality control to identify and correct artifacts or incomplete data before finalizing the scan.

#### 2.3.2. Intraoral Scanners

Two IOS systems were utilized to capture images of all teeth—the Medit i500 (MEDIT Corp., Seoul, Republic of Korea) and Omnicam (Dentsply Sirona, Charlotte, NC, USA). The scan parameters employed were as recommended by the manufacturers, utilizing blue-light mode, a filtering level of 2, and a focal length of 17 mm, in a darkened environment (the dental unit light was switched off). All dental surfaces were dried by air for a five-second duration prior to scanning. The intraoral scans were conducted during the same appointment as the clinical examinations.

Intraoral scanners were calibrated using the manufacturer’s calibration tool and software, in accordance with device-specific protocols. This process is unique to each scanner model. For the Medit i500, calibration was conducted using the Medit calibration tool positioning and the calibration sequence. Intraoral scanning was performed under controlled lighting conditions, with surgical lights deflected to prevent stray illumination. The acquired 3D models were examined on a computer display with a resolution of 1920 × 1080.

The 3D images obtained were displayed using Exocad viewer software (version 1.6.2/2021). Additionally, for direct visual clinical assessment, lesions were evaluated using 3D digital models, and they were classified according to the ICDAS II criteria and documented on an ICDAS chart. There was a three-month delay between the clinical examinations and the recorded ratings whilst visualizing the 3D digital models.

The values allocated by each examiner (rater) on each of the ICDAS-defined surfaces during the examinations were collected in a structured database created in Microsoft Excel for Microsoft 365 MSO (Version 2306 Build 16.0.16529.20164) 64-bit.

### 2.4. Missing Data

The analysis only included complete cases where the values for each ICDAS surface, the examiner, and the assigned identifier of the patient were clearly marked, along with the examiner’s level of expertise and type of examination.

Volunteers with missing information in the ICDAS chart were excluded from all analyses. For 23 patients the ICDAS complete files were recorded for all 3 examinations (clinical and two intraoral scanners). For 7 patients, the ICDAS files were incomplete: 5 of them did not attend all the examination sessions, whereas for 2 patients one or more ICDAS surfaces had incomplete data (only one figure was recorded out of the required 2) for at least one examination.

### 2.5. Statistical Analysis

Agreement between observers with different clinical experiences levels using the same examination technique (inter-rater agreement), as well as agreement for different experience levels observers using different examination techniques (intra-rater agreement) to identify dental restorations were performed for all teeth, or for a group of teeth. From the existing dataset, there were randomly selected cases rated by two highly experienced dentists and two dental students, respectively, during clinical examinations as well as on digital volumetric models acquired with two intraoral scanners for agreement assessment. The raters chosen for statistical analysis were also randomly selected from each experience group. Cohen’s weighted kappa coefficient was applied to assess inter-rater and intra-rater agreement for ordinal data, because for dental restorations (fillings) codes 0–3 can be considered ordinal, whereas codes 3–8 can be considered nominal, therefore prevalence and biased adjusted kappa (PABAK) were also employed. A corresponding *p*-value for agreement was computed for each agreement coefficient. For all statistical tests, a significance level of *p* < 0.05 was used. No adjustment for multiple comparisons was made. No a priori sample size calculations were performed.

Kappa coefficient values were interpreted according to recent literature into the following: <0.00: Poor agreement, 0.00–0.20: Slight agreement, 0.21–0.40: Fair agreement, 0.41–0.60: Moderate agreement, 0.61–0.80: Substantial agreement, 0.81–1.00: Almost perfect agreement [33].

The agreement tests were carried out individually on the anterior (11–13, 21–23, 31–33, 41–43) and posterior teeth (all other teeth, excluding the front teeth) as the visibility and difficulty level of the test varied significantly. To account for this, analyses were made separately for the pits and fissures and for the smooth surfaces.

ICADS categories should be treated as nominal variables because each code represents a distinct, non-ordered category describing the caries process or restoration status, rather than a rank or scale of severity. The statistical methods are appropriate for nominal (categorical) data, such as frequency counts, proportions, or contingency tables, rather than treating the codes as ordinal or continuous variables. This approach is supported by the structure of the ICDAS and is consistent with best practices in categorical data analysis [34]. Treating ICDAS codes as nominal variables avoids inappropriate assumptions about the relationship between categories and ensures valid statistical inference [35,36].

The reporting of this study was conducted in accordance with EQUATOR guidelines (Enhancing the QUAlity and Transparency Of health Research) guidelines, specifically the STROBE (STrengthening the Reporting of OBservational studies in Epidemiology) statement (see Supplementary Material S1) [37,38].

Statistical analyses were conducted employing version 4.3.1 of the R environment for statistical computing and graphics, developed by the R Foundation for Statistical Computing in Vienna, Austria. The irr R package associated to version 4.3.1 of the R environment was used for the analyses [39].

## 3. Results

The examined group consisted of 12 males (M) and 18 females (F), aged between 21 and 34 years old, clinically healthy, with a similar distribution to the male/female ratio in UMFIH dental students. The examiners (raters) group consisted of 36 dental professionals aged between 22 and 46 years old divided into three proficiency levels: (1) eighteen third-year dental undergraduates (7M/11F); (2) ten general dentistry trainees (with 1–3 years’ practical experience): 4M/6F; (3) eight experienced dental professionals (with over 5 years’ clinical experience): 1M/7F. The patient files included at least 144 ICDAS surfaces per patient to give reliable results

Both for Medit i500, as well as for Omnicam, statistically significant intra- and inter-rater agreement between ICDAS codes assigned on digital 3D models and during clinical examination was obtained for experienced dentists (Table 1 and Table 2). The total number of ICDAS surfaces on which the observations were made, both for comparisons in Table 1, as well as for comparisons in Table 2, was 288.

When ICDAS values recorded using Omnicam were compared for examiners with different experience levels, as in the case of senior dentist versus student (for example, examiners number 3 with number 7), there was an inter-rater fair agreement for all teeth of 0.252 Cohen’s kappa with CI 0.195–0.699 and a substantial agreement according to PABAK, but for anterior teeth only, as well as for smooth surfaces only. For posterior teeth, as well as for pits and fissures, the PABAK values showed substantial agreement (Table 3).

For low-experienced raters (dental students), there was also an inter-rater substantial agreement for all teeth, as well as for posterior teeth only according to PABAK’s confidence intervals. When examining digital 3D models acquired with Omnicam intraoral scanner, the agreement coefficient Cohen’s kappa was 0.277 with CI 0.181–0.734 for all ICDAS surfaces (Table 4).

For comparisons in Table 3 and Table 4, the total number of ICDAS surfaces on which the observations were made was 144.

**Figure 1:**
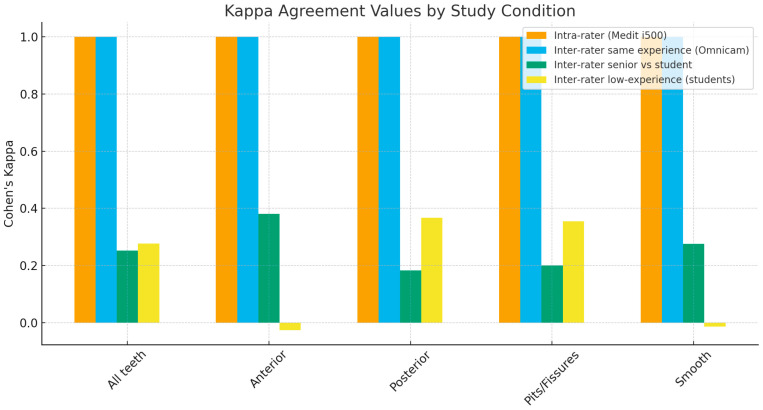
Bar chart of Cohen’s kappa weighted values.

The bar chart of Cohen’s kappa weighted values shows slight inter-rater agreements for senior dentists versus students (Figure 1).

## 4. Discussion

### 4.1. Bias Avoidance Strategy

Applying the ICDAS to fillings and quantifying inter- and intra-rater agreement is part of a larger study we are conducting that aims to train AI software for ICDAS score assessment on 3D virtual models, based on the encouraging results we obtained regarding caries identification [40].

The statistical analysis was performed on randomly selected cases in the dataset so that a uniform image can be obtained:

- intra-rater agreement for different examination methods amongst the 3 methods used, on the same patients;
- inter-rater agreement for the same examination method for different experience levels examiners, tested on the same patient.

We decided to use Cohen’s kappa for ordinal data because the observed values were between 0 and 3—that can be reliably ranked, while 0–8 values cannot be ranked according to the difficulty of visibility.

When values for fillings are 1, 2 or 3, Cohen’s weighted kappa can be used because these values are ordinal (3 being a restoration after a deeper lesion compared to 2, and 2 is more advanced than 1). When there is a “semi-numeric” situation, the PABAK coefficient can be employed, and confidence intervals can be calculated.

The small percentage of tooth-colored restorations may have led to an overfitting (κ = 1) for experienced dentists, due to the limited variability:

- for Medit 500-Clinical, the agreement was 1 probably because there were only nine tooth-colored restorations (code 3 ICDAS), meaning 3.12% out of the total number of surfaces;
- For Omnicam, rater 3 with rater 31, the agreement of experienced raters was also 1, meaning that all nine existing tooth-colored restorations (3.12% of the total number of surfaces) were correctly rated and the surfaces without restorations were identically rated as well.

For Omnicam inter-rater agreement for two raters with a different level of experience (senior dentist versus a 3rd year dental student: observers no. 3 and no. 7) who met only cases with tooth-colored restorations, there were 2 discordances.

Other studies used inter- and intra-rater agreement assessment as a methodology [41].

The present study has several limitations that could be addressed by extending it into a multi-centric study which should also include clinical population. In the present study which was single center, the perfect agreement values (κ = 1.00) are plausible and can be explained by the low variability, limited sample, and simplification of diagnostic categories, and patients and examiners were recruited from the same academic institution (students/interns), which might limit generalizability. This selection bias risk is expected not to influence the findings of this single-centric study in a significant way, because the aim was to identify the intra- and inter-rater agreement on 3D virtual models, especially for cases with very few tooth-colored restorations as a pre-requisite for training AI models. Recent studies have proved that ICDAS criteria can be reliably applied to 3D digital models [42].

All examiners had 30 min of training for each scanner as a strategy to avoid potential intraoral scanning artifacts as a possible source of bias by minimizing the use and extent of cut-off and rescanning procedures, as these have been shown to reduce scan accuracy, particularly when multiple or large areas are rescanned; this ensured that any necessary rescanning does not allow further modification of the preexisting digital scan [43,44,45]. This 30 min training of each examiner was also used in order to avoid bias resulting from impression errors or from the scanning angulation in intraoral scanning, and it includes standardizing the scanning protocol, minimizing rescanning and cut-off procedures, and maintaining consistent scanner angulation. The literature states that standardization of scanning strategy—such as continuous horizontal scanning rather than vertical rotation—reduces trueness errors associated with angulation changes and operator technique [46,47].

- We addressed the memory or learning bias as follows: since the ICDAS pre-calibration, the examiners used the ICDAS daily (in total each examiner filled in up to 90 ICDAS files for this study only). The 3-month interval was between the examinations of the same patient in order for the intra-rater agreement not to be biased by seeing the same patient too soon through different methods. For each patient, the clinical examination and the two intraoral scans were performed on the same day, and then the digital 3D models were examined at 3 months interval to avoid bias due to the same examiner memorizing the ICDAS codes which she/he assigned. Each rater filled in ICDAS paper files without consulting previous scoring of the same patient. Professional cleaning and 5 s of air-drying of all surfaces should have minimized, but not completely eliminated, other possible sources of bias, such as examiner familiarity with ICDAS, scanning artifacts, or differences between scanner types. This 3-month interval between examinations also minimized another possible limitation of the study, the lack of examiner blinding.
- Another possible source of bias could be the experience of the examiner, but one of the aims was to evaluate if despite ICDAS training, the inter and intra-rater agreement depends on the examiner’s experience. This objective was chosen as a groundwork procedure for students and early career dentists’ participation in the validation steps for training AI models for automatic ICDAS-based DMF-S calculation.
- The single-center design provided uniform conditions for examination and access to identical equipment for all raters. Also, in order to minimize ICDAS training variability as a potential source of bias, the training was provided hands-on by the same team of two university lecturers, both of whom are ICDAS-validated trainers.
- The Agency for Healthcare Research and Quality highlights that recall bias is a function of time, and the likelihood of forgetting previous diagnostic experiences increases as the interval between events and evaluation lengthens, especially for less memorable or less impactful findings. While no specific optimal interval is universally established for intraoral scan studies, intervals of several weeks to months are supported by the general principles of minimizing recall bias in diagnostic research [48].
- The low number of tooth-colored dental restorations led to k = 1. We consider that it is realistic for experienced dentists to identify restorations on 3D virtual models, but the fact that early career dental professionals incorrectly rated some of the tooth-colored dental fillings means it can still be an important challenge, for example, to train AI models for automatic dental examination and caries susceptibility assessment. At least the preliminary validation has to be performed by experienced dentists.
- DIAGNOdent exhibits limited reliability in detecting secondary caries due to moderate sensitivity, low accuracy for posterior teeth, and a high risk of false positives, making it suitable only as a supplementary diagnostic aid [49].
- A possible limitation of ICDAS, resulting from systematic reviews and meta-analyses, is that ICDAS is more accurate for secondary caries detection adjacent to composite restorations than amalgam [50]. However, we addressed this possible issue, including only patients without amalgam restorations.

### 4.2. Future Perspectives

Based on the available data, the present study can be extended to check if DIAGNOdent scores are correlated to dental restoration material (glass-ionomer or composite—first figure ICDAS = 3).

Remineralization options depend on early detection of secondary caries. Artificial Intelligence (AI) could be used for caries risk assessment based on patient’s past caries experience, including the number of dental restorations.

Any caries complications may lead to more expensive and complicated treatments, even from the legal point of view [51].

Stakeholders have already tried AI-assisted caries recognition, but only beta-versions were briefly available [52]. Also previous studies showed reliability of digital volumetric models in cariology [14,15].

The novelty of our study lies in the initial steps taken toward measuring the presence (material codes) or absence of dental fillings on digital 3D models, serving as a reference point for the objective assessment of caries susceptibility [53].

It could also be extended to check inter- and intra-rater agreements for secondary caries: first figure of ICDAS code >0 meaning thatthere are fillings on the same surface/area and second figure of the ICDAS code > 0; second test for early stage secondary caries: first figure of ICDAS code >0 meaning thatthere are fillings on the same surface/area and second figure of the ICDAS code >1 and ≤3.

Knowing that men and women have slight differences in perceiving colors, it could be assessed whether there are any inter- and intra-rater agreements differences between male and female students.

Tele-dentistry tends to be a common approach and picture/images-based oro-dental examination will be among mandatory dentist skills [54].

Machine learning models are also being tested, as well as pilot studies for robot-assisted treatment [55].

AI-based caries diagnosis and management is slowly becoming possible [56]. In 2021 a study that was published in Nature Scientific Reports, had clinically evaluated the performance of an automated occlusal caries assessment tool [14]. A notable finding was that an intraoral scanner using transillumination together with DIAGNOcam provided reliable detection for proximal caries, whereas standard diagnostic approaches were less effective for identifying proximal lesions [15]. There are also studies that investigated the intraoral scanning for assessing the gingival morphological changes [16]. Some limitations from the IOS studies highlight the need for further development, larger and more diverse training datasets, and improved algorithms to enhance the reliability and clinical utility of automated dental filling detection tools. Previous research has examined the reliability of intraoral scanning for the detailed detection of dental preparations intended for prosthetic restorations, such as onlays and inlays. These studies have also emphasized the technical challenges associated with accurate margin capture and have proposed various strategies to address these limitations [57,58,59]. Intraoral scanning has been tested in vitro on typodont models to assess the accuracy of detecting primarily indirect restorations made from various materials, as well as certain direct composite restorations [60].

Building upon this promising background, the present in vivo study focused on evaluating the accuracy of intraoral scanning in detecting existing dental restorations. This approach aims to contribute to the development of individualized preventive care strategies and to enhance the precision of patient-specific treatment planning within digital dentistry workflows.

Accordingly, training AI models for automatic recognition of fillings and new caries could be a next step for faster image-based diagnosis and better motivation of the patients.

The most appropriate agreement statistic for this study was Cohen’s kappa, and the targeted confidence interval width for sample size calculation was 0.20 for the 95% interval [61,62,63]. Within this framework, the minimum sample size required to reliably evaluate inter-rater and intra-rater agreement for ICDAS-assessed tooth surface—under conditions involving 36 raters of three experience levels and the use of two intra oral scanners in addition to clinical examination—comprises 10 patients and a minimum of approximately 700 tooth surfaces. These thresholds are substantiated by prior reliability investigations reporting consistently high kappa coefficients and reproducible outcomes when 10 patients and roughly 700–705 surfaces were evaluated by multiple raters using ICDAS criteria [61]. Furthermore, studies with comparable calibration and reliability objectives have employed sample sizes ranging from 10 to 20 and 700 to 3600 surfaces to produce stable and dependable agreement estimates, even when incorporating large examiner cohorts and multiple assessment modalities [61,64]. Although increasing the number of evaluated surfaces beyond 700 may enhance the precision of agreement estimates—particularly when stratifying analyses by examiner experience or diagnostic modality—the existing ICDAS literature indicates that a sample such as ours, including at least 144 ICDAS surfaces per patient, gives reliable results [61].

## 5. Conclusions

Inter- and intra-rater agreement on fillings identification on 3D digital models obtained by intraoral scanning was substantial in most analyses in our study.

This consistency suggests that intraoral scanning provides reliable and reproducible diagnostic information. Such reliability supports its practical value as a standardized tool for dental restorations assessment, facilitating the consistent detection and documentation of dental fillings across clinicians. In turn, this may reduce reliance on radiographic imaging and thus minimize patient exposure to ionizing radiation. Furthermore, these findings align with the principles of minimally invasive dentistry and encourage broader integration of digital workflows into diagnostic and treatment planning processes, even AI validation for secondary caries detection.

Although two intraoral scanner models were used to enhance the generalizability of the results and clinical examination was included for comparison, several limitations should be acknowledged. The study’s sample size and controlled experimental conditions may not fully represent the variability encountered in everyday clinical practice.

Differences in scanner calibration, scanning algorithms, and software processing among various systems may also influence diagnostic outcomes. Additionally, the absence of radiographic verification limits the ability to draw definitive conclusions regarding diagnostic sensitivity and specificity. Nevertheless, ICDAS-based DMF-S quantification on digital 3D models brings an important clinical significance by helping to determine how frequently patients should attend regular dental check-ups, professional cleaning, and local fluoridation based on past caries history (DMF-S).

Future research involving larger and more heterogenous groups of patients, multiple scanner systems, and additional diagnostic modalities is recommended to further validate and expand the potential of intraoral scanning as a standardized framework for restorations assessment.

## Figures and Tables

**Table 1:** Intra-rater agreement for ICDAS fillings scores assigned on Medit i500^®^ scanned images with clinical examination.

Observations	Number of Observations	Cohen’s Kappa Weighted	95% CI	*p*-Value	PABAK 95% CI
All teeth	288	1	(1–1)	<0.001	1 (0.975–1)
Anterior teeth	104	1	(1–1)	<0.001	1 (0.93–1)
Posterior teeth	184	1	(1–1)	<0.001	1 (0.96–1)
Pits and fissures	64	1	(1–1)	<0.001	1 (0.888–1)
Smooth surface	224	1	(1–1)	<0.001	1 (0.967–1)

CI, confidence interval; PABAK, prevalence and bias adjusted kappa; significance level *p* < 0.05.

**Table 2:** Inter-rater agreement for ICDAS fillings scores assigned on Omnicam scanned images with clinical examination for examiners with the same experience level (senior dentists).

Observations	Number of Observations	Cohen’s Kappa Weighted	95% CI	*p*-Value	PABAK 95% CI
All teeth	288	1	(1–1)	<0.001	1 (0.975–1)
Anterior teeth	104	1	(1–1)	<0.001	1 (0.93–1)
Posterior teeth	184	1	(1–1)	<0.001	1 (0.96–1)
Pits and fissures	64	1	(1–1)	<0.001	1 (0.888–1)
Smooth surface	224	1	(1–1)	<0.001	1 (0.967–1)

CI, confidence interval; PABAK, prevalence and bias adjusted kappa; significance level *p* < 0.05.

**Table 3:** Inter-rater agreement for ICDAS fillings scores assigned on Omnicam for examiners with different experience leves: senior dentist versus student.

Observations	Number of Observations	Cohen’s Kappa Weighted	95% CI	*p*-Value	PABAK 95% CI
All teeth	144	0.252	−0.195–0.699	0.269	0.861 (0.752–0.932)
Anterior teeth	52	0.381	−0.299–1.061	0.272	0.885 (0.681–0.976)
Posterior teeth	92	0.183	−0.399–0.765	0.538	0.848 (0.699–0.938
Pits and fissures	32	0.2	−0.444–0.844	0.543	0.688 (0.344–0.894)
Smooth surface	112	0.275	−0.347–0.896	0.386	0.911 (0.798–0.971)

CI, confidence interval; PABAK, prevalence and bias adjusted kappa; significance level *p* < 0.05.

**Table 4:** Inter-rater agreement for fillings scores assigned on Omnicam 3D images for low-experience level examiners.

Observations	Number Observations	Cohen’s Kappa Weighted	95% CI	*p*-Value	PABAK 95% CI
All teeth	144	0.277	−0.181–0.734	0.236	0.875 (0.769–0.942)
Anterior teeth	52	−0.026	−1.154–1.101	0.964	0.885 (0.681–0.976)
Posterior teeth	92	0.367	−0.123–0.857	0.142	0.87 (0.727–0.951)
Pits and fissures	32	0.355	−0.165–0.874	0.181	0.688 (0.344–0.894)
Smooth surface	112	−0.014	−0.989–0.962	0.978	0.929 (95% CI 0.822–0.98)

CI, confidence interval; PABAK, prevalence and bias adjusted kappa; significance level *p* < 0.05.

## Data Availability

Data will be made available upon request due to privacy and ethical restrictions. The details regarding where data supporting reported results can be sent upon request from corresponding authors or last author. No publicly archived datasets were analyzed. Some data might be unavailable due to privacy or ethical restrictions.

## Supplementary Materials

The following supporting information can be downloaded at: https://www.mdpi.com/article/10.3390/medicina61122135/s1, STROBE Statement—Checklist of items that should be included in reports of cross-sectional studies.

## Abbreviations

The following abbreviations are used in this manuscript:

3D	Three-Dimensional
AI	Artificial Intelligence
CBCT	Cone-Beam Computed Tomography
DMFs	Decayed, Missing, and Filled Surfaces
EQUATOR	Enhancing the QUAlity and Transparency Of health Research
ICDAS	International Caries Detection and Assessment System
IOS	Intraoral scanning
LF	Laser Fluorescence technique (DIAGNODent 2095 (LF), KaVo, Biberach, Germany)
PABAK	Prevalence and biased adjusted kappa
STROBE	STrengthening the Reporting of OBservational studies in Epidemiology
VR	Virtual Reality
WHO	World Health Organization
